# Periscope of Dermatology

**Published:** 1894-11-03

**Authors:** 


### Periscope of Dermatology.

**Parasitic Diseases.**—Wickham[1] recommends energetic treatment in the last stage of tinea, and employs either electrolysis, the cautery, or croton oil. In this way the last stage can be shortened by several months. Belère[2] concludes, from microscopic examinations, that there are two distinct varieties of trichomycosis which are caused by different parasites, the one being characterised by the presence of small spores arranged in a mosaic round the hair, the other by the existence of large chain-like spores within the interior of the hair. The large-spored variety is alone a trichophyton, the other being more properly called a trichomycosis. He further found that the large-spored fungus included several other varieties. The *Medical Week*[3] reviews a graduation thesis upon this subject by Dr. Martin, in which reference is made to all the different forms of parasite described by Sabouraud and others, and an appropriate treatment is suggested for each variety. Purdon[4] records a case of Burmese ringworm, or eczema marginatum, which occurred in a soldier who had been in India. The disease, which had commenced four years before, consisted of well-defined rings on the buttocks, the thighs, and lumbar regions, and recurred after apparent cure. The disease is supposed to be confined to the sea-coast of India, especially that of Burma and Malabar; it is rare in females. Adamson[5] reviews the work of Sabouraud upon the various kinds of trichophyton; he considers that about one-half of the cases of tinea tonsurans are due to the microsporon audouini, which was first described by Gruby as the cause of porrigo decalvans. This organism is distinct, both microscopically and by cultivation experiments, from that of tinea tonsurans; it is not a trichophyton.

**Onychomycosis.**—Sabrazès[6] found that the fungus obtained from trichophytosis of the nails is identical with the fungus of favus, and produces characteristic cup-shaped scales when inoculated on the ears of mice. Abraham and Eddowes[7] recommend the periodic inspection of the heads of school children by skilled observers, in order to detect early cases of ringworm and impetigo. They also suggest that a greasy germicide ointment should be applied while the child attends school. Savill[8] describes a skin disease occurring on the face of school children, which, though resembling ringworm in appearance, did not affect the hairy parts. He considered the condition allied to the epidemic skin disease described by him in 1891.

**Acarophobia.**—Thibierge[9] states that persons who are pursued by a fear of itch are a very difficult class to treat; they are manifestly neurotic, and describe a symptomology more or less allied to itch. In those who have had a previous attack, the persistence of itching for some time after the disease is cured will account for the condition. In others who have never had the disease, but who have run the risk of contagion, any pruriginous eruption is firmly believed to be the itch. In a last class of cases the condition is due to cocainomania, where a particular form of delirium is observed; the patient experiences peculiar sensations, and continually wants to prick his skin with a needle in order to remove imaginary foreign bodies, which he believes to be acari. In all these cases the treatment consists in the inunction of a 10 per cent. napthol ointment after a hot bath with plenty of soap.

**Scleroderma.**—Patteson[10] describes a case of circumscribed scleroderma occurring in a patient twenty-eight years of age. It consisted of two raised yellow patches, one on the forehead and another small one on the cheek; a third spot subsequently appeared exactly over the mental foramen. The distribution of these patches of affected skin was in relation to the branches of the fifth nerve, the starting point of each being the point of exit of a branch through a bony foramen. Du Castel[11] showed a patient affected with scleroderma, limited to the left side of the chest, and in which the sclerotic patches had a manifest zosteriform arrangement. In drawing attention to the resemblance of this disease to leprosy, he stated that the diagnosis would be established by a bacteriological examination of the fluid obtained from a blister. Boisseau du Rocher[12] describes the case of a woman, aged forty-six years, who for a year had suffered from scleroderma of the dorsum of the foot, and had been treated by static electrical discharges of high potential and powerful delivery by a special apparatus. A rain of painless sparks was directed on the affected skin, which became reddened, and which subsequently desquamated, leaving a healthy new skin as a result.

**Psoriasis.**—Shoemaker[13] considers that psoriasis is due to several different internal conditions, and so can best be treated by internal remedies. The diseases which most commonly are associated with it are rheumatism, gout, chronic disturbance of the digestion, and diseases of the liver, the blood, or nervous system. The particular causative disease must be diagnosed in each case, and an appropriate internal treatment carried out. Thibierge[14] refers to a case of psoriasis where the eruption followed the course of the long saphena, the musculo-cutaneous, and the small sciatic nerves of one side. He considers that this proves the nervous origin of the disease in some cases. Another case was referred to where psoriasis was confined to the distribution of a nerve in the arm, and a third case where a pustular eruption corresponded to the course of the ulnar nerve, which had suffered an injury.

**Seborrhœa.**—Beatty[15] comments upon Unna's views on the origin of this disease, notice of which has appeared in a former review of The Hospital. He, however, differs from Unna in not considering that oily seborrhœa is a hypersecretion of the sweat glands.

His observations defend the old-established view that seborrhœa is a disease of the sebaceous glands, and that the sweat glands do not secrete fat. On the other hand he confirms Unna's view that the so-called dry seborrhœa is an inflammatory condition, and may therefore be included under the general term of seborrhoic eczema. Payne[16] also doubts the truth of Unna's teaching, especially with regard to the specific cause of eczema seborrhoica. However, he admits the relation between seborrhœa and eczema. For treatment he prefers an ointment containing sulphur, and acid carbolic, a.a. grains 15 vaseline, ℥1. This is to be rubbed into the scalp after it has been thoroughly disinfected by lotions of perchloride of mercury.

**Lichen.**—Patteson[17] describes three cases of lichen ruber planus, in all of which the eruption was limited to the legs, and did not extend much beyond the knee. The papules were of a red colour, sharply defined, and arranged in an interlacing manner; the itching was intense, being worse at night, but was relieved by a two per cent. solution of carbolic acid, together with arsenic internally. Meneau[18] investigates the relationship of lichen pilaris to congential icthyosis, and attributes the former disease to hereditary predisposition. Lichen pilaris is usually more or less marked in most cases of icthyosis, so he regards the disease as an attenuated form of icthyosis which is localised in the hair follicles. The alopecia due to this disease is incurable when once the lesions are established.

**Pigmentary Diseases.**—Meynan[19] relates the case of a child who, at birth, presented extensive pigmentation of the back. It had started from a V-shaped patch over the spine, which was absolutely black, but otherwise normal in texture. No reason could be given for the presence of this pigmentation. Morris[20] showed at the Royal Medico-Chirurgical Society a case of acanthosis nigricans. It occurred in a single woman aged 35 years, who for the last nine months had noticed some bronzing of her skin; crops of flat warts then appeared on the hands, the axillæ, the umbilicus, and elsewhere. Later black patches appeared, which were most marked near the warts. Nothing is known of the pathology of this condition, but improvement followed the use of salicylic acid.

**Eruptions Due to Drugs, &c.**—Fournier[21] describes an eruption on the palms of the hands, resembling an ordinary syphilide, which appeared within a few hours of taking eight grains of antipyrine. The patient had previously suffered from syphilis, but this eruption quickly disappeared, and occurred again after a similar dose of the drug. Morton[22] describes an eruption of dark red erythematous spots the size of a split pea, which occurred on the trunk and lower limbs during an attack of gonorrhœa treated with sandal wood oil. The eruption disappeared a few days after the drug was stopped, so he considers that the rash was due to the sandal wood oil and not to the original disease. Stavely[23] draws attention to a scarlet rash following the use of enemata, and even brisk aperients when given for chronic constipation; he has noticed it, too, in the later stages of acute intestinal catarrh. In all these cases indican was present in the urine. There is intense itching and tingling, but the rash is not urticarial. Walsh[24] compares this form of rash to those following the injection of Koch's tuberculin. Singer[25] attributes the production of many cutaneous affections to intestinal fermentation, and so considers that in many cases of skin disease intestinal asepsis should be secured by the administration of capsules of menthol and olive oil. Colcott-Fox[26] records an urticarial eruption which appeared in a case of acute osteomyelitis, and states that such rashes are now well recognised in any case of toxæmia; their early appearance may easily cause the disease to be confounded with rheumatism.

**Therapeutics.**—Thiol[27] is recommended by Rudneff for the treatment of erysipelas; a 20 to 40 per cent. watery solution should be painted on to the part five times a day. Most of the cases were cured within two to four days, but the drug has disadvantages, since it stains linen and is very costly. It is, however, odourless, and has no toxic properties. Next to thiol, a 1 in 500 solution of mercuric chloride is the most efficacious application. Hodara[28] recommends the following form of glycerine jelly as the best for ordinary purposes; it melts at the temperature of 100 and sets at 82 Fahr.: ℞. water 55 parts, gelatine 12·5 parts, glycerine 12·5 parts, zinc oxide 20 parts. Mix in water bath. Taenzer[29] finds that Unna's plaster muslins retain their activity and adhesiveness even after the lapse of many years; he has found the salicylic plaster useful for hyperkeratoses, warts, chronic eczema, and scleroderma. The mercuric plaster has quite superseded inunction in the treatment of syphilis; the pyrogallol plaster, too, is useful for lupus erythematosus. Statical electricity is recommended by Dounier[30] for both acute and chronic eczema; he advises the use of a machine giving sparks three inches long and an electrode with either a breeze or a brush discharge.

**New Growths.**—Fordyce[31] showed a specimen of adeno-carcinoma of the skin, which clearly showed its origin from the sweat glands; the tumour was removed from the leg of a man aged thirty-five years. White[32] described a condition which he names angioma serpiginosa. It consisted of twenty-four lesions the size of a pin's head, which were situated between the scapula and the left nipple. The colour was red, and did not disappear under pressure. The eruption spread in an annular manner for an indefinite time, while the central parts underwent evolution. Pathologically the disease has a great resemblance to pigmented moles. Coleman[33] describes a case of osteoma of the skin; it occupied one-third of the plantar surface of the foot of a child aged six years. The plaque was perfectly moveable over the deep parts, and had existed for two and a half years. Microscopically it contained spicules of true bone, together with some cartilage and fibrous tissue; typical ossification was progressing at several points.

**Miscellaneous.**—Patëzon[34] observed stripes and furrows on the nails of a man, aged sixty, who suffered from attacks of dermatitis exfoliativa. The separations between the ridges corresponded to the respites from the disease; the length of these healthy portions of nail bore a constant relation to the length of time that he was free from the disease. The medico-legal importance of these traces of previous illness is apparent.

Montgomery[35] reports the case of a young man who had suffered from signs of leprosy for seven years. Sections of a leprous patch on the face were made, but though no bacilli were found, giant cells, in whose periphery was a single row of round nuclei, were abundant.

Riedel[36] has found elephantiasis and œdema to follow the excision of enlarged inguinal glands. It occurred in two cases, and followed repeated attacks of erysipelas. He therefore doubts the advisability of completely removing the inguinal glands.

The diascope[37] is the name given by Unna to a piece of curved glass with raised edges, which has been introduced into dermatology with the object of studying the effects of colouration in various skin affections. He claims that by its use he can discover the yellow cellular elements within the exsanguineous white areolar tissue, and can exactly determine the quantity and seat of the intra-vascular blood; he can also distinguish between a transudation and an exudation. Thus he finds that he can distinguish between œdema and cell infiltration, also between epidermic bullæ and dermal bullæ.

Dermographismus[38] is the term applied to a peculiar condition of the skin occurring under various circumstances, but depending for its appearance on mechanical irritation. In the common form of the disease small raised œdematous patches appear round the roots of the hair; on stroking the skin, too, an isolated group of white papules appear, which give rise to considerable irritation. Erhman draws a distinct difference between dermographismus and urticaria, since the latter disease is caused by some toxic absorption, while the former is produced by the influence of the nervous system alone.

[1] Med. Week, June 22, 1894, p. 298. [2] Ditto, ditto, p. [3] Ditto, June 29, p. 310. [4] Dub. Med. Journ., June-July 2, 1894, p. 21. [5] Brit. Journ. of Derm., June 5, 1894, p. 188. [6] Med. Week, July 13, 1894, p. 330. [7] Lancet, Aug. 4, 1894, p. 275. [8] Ditto, June 30, 1894, p. 1,634. [9] New York Med. Journ., May 19, 1894, p. 638. [10] Dub. Med. Journ., June 1, 1894, p. 537. [11] Med. Week, June 22. 1894, p. 297. [12] Practitioner, June, 1894, p. 453. [13] Med. Record, July 7, 1894, p. 24. [14] Fortsch. Der Med., July 13, 1894, p. 555. [15] Brit. Journ. of Derm., June, 1894, p. 161. [16] Dub. Med. Journ., June 1, 1894, p. 522. [17] Ditto, July 2, 1894, p. 45. [18] Med. Week, June 22, 1894, p. 298. [19] Lancet, July 7, 1894, p. 20. [20] Med. Week, June 15, 1894, p. 281. [21] New York Med. Journ., June 30th, 1894, p. 831. [22] Brit. Journ. of Derm., June, 1894, p. 171. [23] Brit. Med. Journ., June 30, 1894, p. 1413. [24] Ditto, July 7, 1894, p. 12. [25] Lancet, Aug. 4, 1894, p. 255. [26] Ditto, Aug. 4, 1894, p. 255. [27] Brit. Med. Journ., June 30, 1894, p. 103. [28] Ed. Med. Journ., Aug., 1894, p. 185. [29] Ditto, ditto, p. 186. [30] Brit. Med. Journ., Ep., July 14, 1894, p. 8. [31] Med. Record, July 21, 1894, p. 86. [32] Ditto, ditto, p. 87. [33] Ditto, Aug. 11, 1894, p. 187. [34] Lancet, July 28, 1894, p. 230. [35] Int. Med. Mag., June, 1894, p. 384. [36] Brit. Med. Journ., Ep., Aug. 4, 1894, p. 18. [37] Brit. Journ. of Derm., June, 1894, p. 186. [38] Lancet, June 30, 1894, p. 1634.

